# Research on establishment of bonded particle model for pellet feed and its application

**DOI:** 10.1038/s41598-026-43893-y

**Published:** 2026-03-12

**Authors:** Zhaoxia Liu, Xianrui Kong, Weixia Wang, Huimei Liu, Bing Leng, Jian Zhou

**Affiliations:** 1 College of Mechanical and Electrical Engineering, Wuhan Donghu University, Wuhan, 430212 China; 2 College of Engineering, Huazhong Agricultural University, Wuhan, 430070 China; 3 Chinese Academy of Agricultural Mechanization Sciences Group Co., Ltd., Beijing, 100083 China

**Keywords:** Pellet feed, Bonded particle model, Parameter calibration, Discrete element method, Breakage characteristics, Engineering, Materials science

## Abstract

This study develops and validates a bonded particle model to investigate impact-induced breakage of piglet pellet feed using the discrete element method. To address the lack of reliable DEM parameters for pellet attrition, bonding properties were calibrated through uniaxial compression experiments combined with numerical simulations and response surface optimization. The calibrated parameters include a normal stiffness per unit area of 2.00e10 N/m^3^, a tangential stiffness per unit area of 7.41e9 N/m^3^, and bond strengths of 500 MPa. The validated model was applied to simulate pellet motion and fragmentation in a centrifugal impact device. The breakage rate increased monotonically with impeller speed from 500 to 1500 rpm, reaching 21.43% at the highest speed, in good agreement with experimental results. A strong linear correlation (R^2^ = 0.9970) between simulated bond breakage fraction and experimentally measured mass-based breakage rate confirms that bond failure percentage provides a physically meaningful descriptor of macroscopic fragmentation. In addition, breakage exhibited a non-linear dependence on impact angle, with a maximum value of 6.86% at 75° due to the combined axial and radial loading effects. The proposed framework can provide theoretical guidance for improving pellet durability and optimizing processing conditions.

## Introduction

Pellet feed is widely used in aquaculture and livestock production because of its balanced nutritional composition, convenient storage and transportation, reduced selective feeding, and high digestibility^1^. The pellet durability index (PDI) is a key parameter for evaluating the processing quality of pellet feed, as it reflects the resistance of pellets to mechanical degradation. Specifically, PDI quantifies the proportion of fines and powder generated when pellets are subjected to vibration, impact, friction, and compression during storage, transportation, and handling^2,3^. Pellet breakage is therefore unavoidable in practical applications. Excessive fine generation not only diminishes the advantages of pellet feed and reduces feed utilization efficiency, but may also adversely affect animal health and environmental quality^4–6^. In addition, poor pellet quality increases material loss during transportation. Consequently, a comprehensive understanding of pellet degradation mechanisms is essential for optimizing feed production, reducing manufacturing costs, improving feeding efficiency, and promoting the sustainable development of aquaculture and livestock industries^7,8^.

Early investigations of pellet breakage primarily relied on conventional experimental methods^9–11^. In recent years, the Discrete Element Method (DEM) and the coupled Computational Fluid Dynamics–Discrete Element Method (CFD–DEM) have been increasingly employed to analyze the mechanical behavior of pellet feed. These numerical approaches enable detailed examination of particle interactions, collision processes, and breakage phenomena, thereby providing valuable insight into the mechanisms governing pellet degradation^12–14^. However, owing to the diversity of pellet feed compositions and geometrical structures, accurate calibration of DEM parameters remains essential to ensure the reliability and predictive capability of numerical simulations.

Significant efforts have been devoted to the calibration of DEM parameters for agricultural materials, resulting in two main strategies: bulk calibration and direct measurement methods^15^. The bulk calibration approach reproduces experimentally observed macroscopic behavior by iteratively adjusting DEM parameters in numerical simulations, whereas the direct measurement approach determines material and contact parameters through particle-scale experiments^16^. Using techniques such as angle of repose tests, inclined plate impact tests, and parameter optimization methods, key parameters—including intrinsic material properties, friction coefficients, and restitution coefficients—can be systematically determined. With appropriate calibration, DEM models are capable of reproducing the kinematic and stress responses of agricultural materials under realistic operating conditions^17–19^. Nevertheless, conventional multi-sphere DEM models exhibit limited capability in representing pellet breakage states and internal energy evolution, such as cohesion energy density^20^. This limitation underscores the need for an advanced simulation framework that can more effectively capture pellet feed breakage mechanisms.

To address these limitations, the present study focuses on the development and calibration of a pellet feed breakage model. A bonded particle model based on the Hertz–Mindlin contact law with bonding is established. The bonding parameters are calibrated through a combination of uniaxial compression experiments and corresponding DEM simulations, supported by experimental design and optimization techniques. The validated model is subsequently applied to simulate pellet breakage in a centrifugal impact tester, providing mechanistic insight into pellet degradation under dynamic loading conditions.

## Materials and methods

### Experiment material

**Fig. 1:**
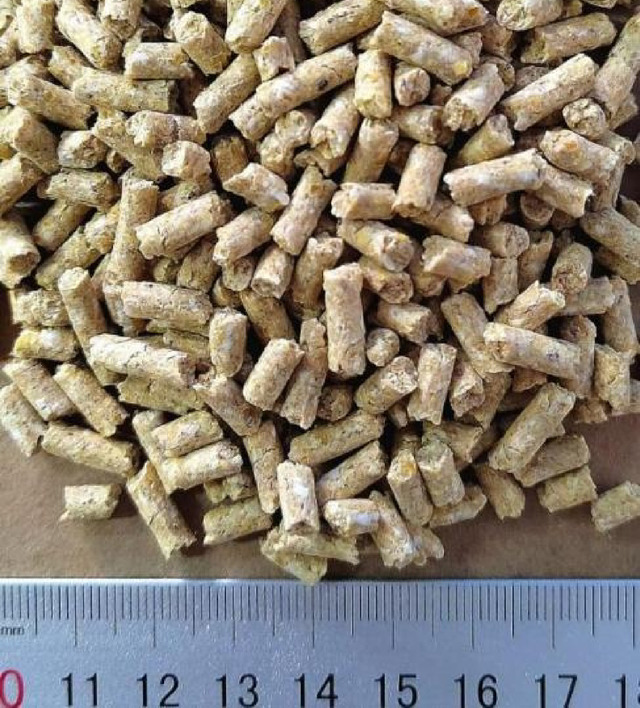
Specimens of pellet feed.

The samples were obtained from a commercial feed production enterprise (New Hope Liuhe Co., Ltd., Huanggang, China). The material consisted of post-weaning piglet compound feed, manufactured from corn, soybean meal, wheat, and wheat bran through standard processing steps including ingredient dosing, grinding, mixing, and pelleting. A representative sample is presented in Fig. 1. For dimensional characterization, 100 pellets were randomly selected. Pellet length and diameter were measured using a digital vernier caliper (Guanglu Digital Measurement & Control Co., Ltd., Guilin, China) with an accuracy of 0.01 mm. The average pellet length was 12.47 ± 1.22 mm, and the average diameter was 4.16 ± 0.08 mm. Pellet mass was measured using an electronic balance, and pellet volume was determined by the sand displacement method. Based on these measurements, the average pellet density was calculated as 1093.28 ± 21.83 kg/m^3^. The moisture content was determined using the oven-drying method according to GB/T 6435–2014. Five parallel samples were analyzed, yielding an average moisture content of 12.30 ± 0.03%.

### Uniaxial compression test

**Fig. 2:**
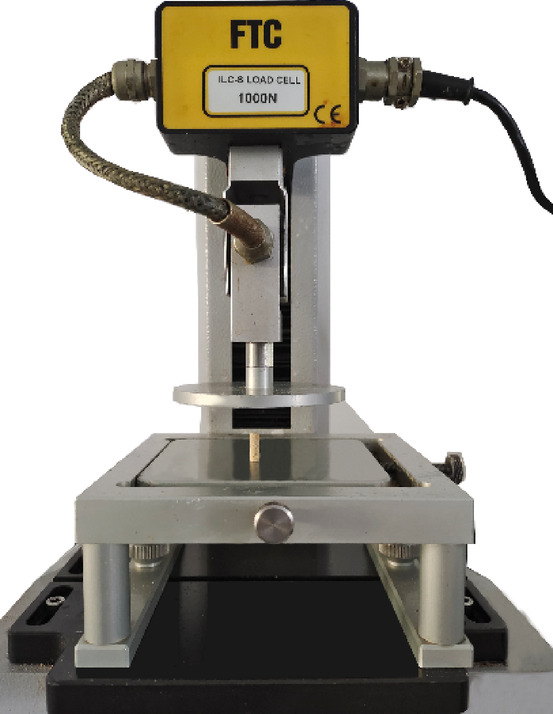
Uniaxial compression test of pellet feed.

Uniaxial compression tests were performed using a TMS-Pro texture analyzer (FTC, USA) to obtain the breakage information. A schematic of the experimental setup is shown in Fig. 2. Prior to testing, the pellets were gently polished using 320-grit sandpaper to standardize their length to 12.47 ± 0.10 mm. During the tests, the compression speed was set to 10 mm/min, and the axial deformation was set to 3 mm. The elastic modulus was calculated from the changes in pellet height and diameter measured before and after the compression tests, yielding an average value of 258.83 ± 61.58 MPa. The corresponding Poisson’s ratio was determined to be 0.34 ± 0.06. Based on the tests conducted on 100 pellets, the average breakage force was 32.3 ± 6.5 N.

### Simulation model establishment

#### Bonded particle model of pellet feed

**Fig. 3:**
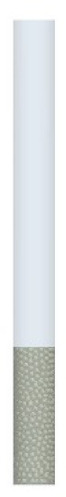
Establishment of bonded particle model.

A cylindrical particle factory was constructed in EDEM software, with its diameter set equal to the average diameter of the pellet feed (4.16 mm) and its height slightly greater than the average length. Considering both computational efficiency and the resolution required to represent fines generation, spherical particles with a radius of 0.4 mm were generated to fill the particle factory. The particle factory was initialized in a static state, and particle filling was carried out using the Hertz–Mindlin contact model. The filling process continued until the velocities of all particles decreased below 1 × 10^− 5^ m/s, indicating a stable packing state. Subsequently, particles located outside the target pellet geometry were removed, resulting in a filled pellet model composed of 300 spherical particles, as illustrated in Fig. 3. To ensure consistency between the numerical model and the actual pellet mass, a density scaling approach was employed^21^. Specifically, the density of the spherical particles was increased from 1093.28 kg/m^3^ to 2305.86 kg/m^3^, while all other parameters remained unchanged. The contact radius between particles was set to 0.44 mm^22^. Finally, the contact model was switched to the Hertz–Mindlin with bonding, and the corresponding bonding parameters were assigned to establish the bonded particle model (BPM) of the pellet feed.

The bonding parameters in the bonded particle model (BPM) mainly include the normal stiffness per unit area, tangential stiffness per unit area, normal bond strength, and tangential bond strength^23^. To improve the efficiency and robustness of the calibration procedure, several analytical expressions for estimating the normal and tangential stiffness of bonded contacts have been proposed in previous studies^24–26^. The expressions for the normal stiffness and tangential stiffness are given as follows:

1 $${k_n}=\frac{{{E_c}}}{L}$$

2 $${k_s}=\frac{{{G_c}}}{L}$$

3 $${E_c}=2{G_c}(1+\nu )$$

4 $$L={R_1}+{R_2}$$

where *k*_n_ and *k*_s_ are the normal stiffness per unit area and tangential stiffness per unit area of the bond, N/m^3^; *E*_c_ and *G*_c_ are the contact elastic modulus and contact shear modulus, MPa; *ν* is the Poisson’s ratio of the sphere; *L* is the length of the bond, m; *R*_1_ and *R*_2_ are the radii of sphere 1 and sphere 2, mm.

This study adopts a combined calibration strategy integrating analytical estimation and numerical simulation to determine the bonding parameters of the BPM. According to the above formulations, the ratio of the normal stiffness to the tangential stiffness can be expressed as 2(1 + *ν*). Substituting the Poisson’s ratio of the pellet feed yields a proportionality coefficient of 2.68. Based on preliminary simulation tests and in reference to existing studies^13,14,27–29^, the bonding parameters assigned to the pellet feed are listed in Table 1.

**Table 1:** The range of bonding parameters.

Parameter	Value
Normal stiffness per unit area *x*_1_/(N/m^3^)	1 × 10^10^~5 × 10^10^
Tangential stiffness per unit area *x*_2_/(N/m^3^)	3.73 × 10^9^~1.87 × 10^10^
Normal strength *x*_3_/MPa	100 ~ 900
Tangential strength *x*_4_/MPa	100 ~ 900

#### Simulation model set-up

**Fig. 4:**
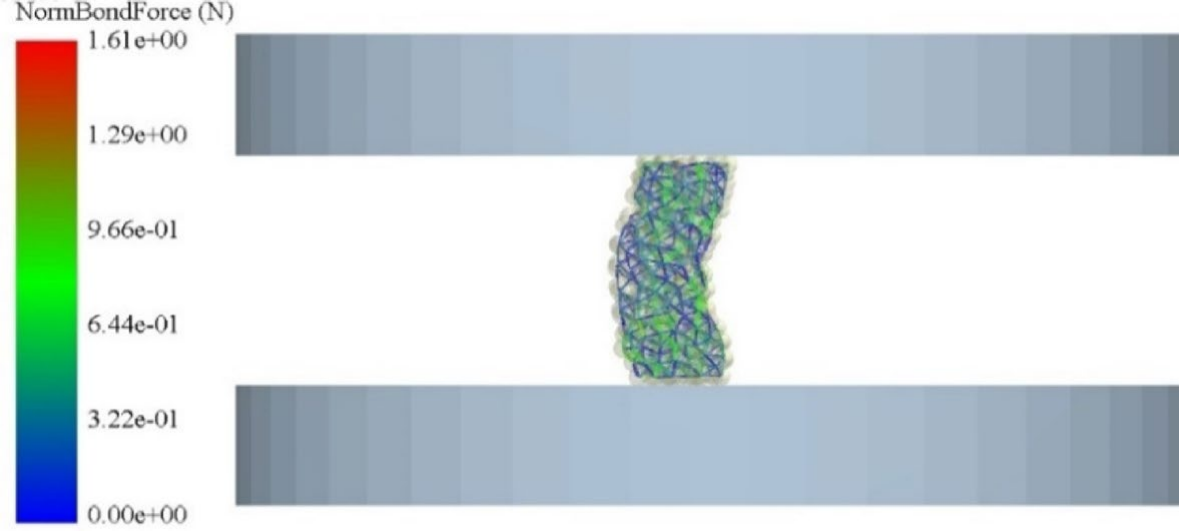
Simulation model of Uniaxial compression test.

A numerical model of the uniaxial compression test was established based on the actual geometries of the compression and base plates. Geometric details with negligible influence on the mechanical response were omitted, yielding the simplified model shown in Fig. 4. The Rayleigh time step was calculated as 6.23 × 10^− 6^ s. A numerical time step of 4.00 × 10^− 7^ s (6.42% of the Rayleigh limit) was adopted to ensure stable integration. The upper plate was driven downward at 10 mm/min, consistent with experimental conditions. Simulations were performed for 30 s, with data recorded every 0.10 s.

#### Calibration of bonding parameters

Preliminary sensitivity analysis and previous studies^13,14^ indicated that, under quasi-static compression, breakage force is stiffness-dominated. When bond strengths were varied between 100 and 900 MPa, the breakage force changed by less than 0.67%. Therefore, normal and tangential bond strengths were fixed at 500 MPa to reduce parameter non-uniqueness. The normal stiffness per unit area and tangential stiffness per unit area significantly affected the force-displacement response and were calibrated using Response Surface Methodology with a Central Composite Design (CCD). The coded parameter levels are listed in Table 2.

**Table 2:** Coding of bonding parameters.

Coding	Parameters	
	x_1_/(*N*/m^3^)	x_2_/(*N*/m^3^)
−1.414	0.17 × 10^10^	0.63 × 10^9^
−1	1.00 × 10^10^	3.73 × 10^9^
0	3.00 × 10^10^	1.12 × 10^10^
1	5.00 × 10^10^	1.87 × 10^10^
1.414	5.83 × 10^10^	2.18 × 10^10^

### Application of BPM

**Fig. 5:**
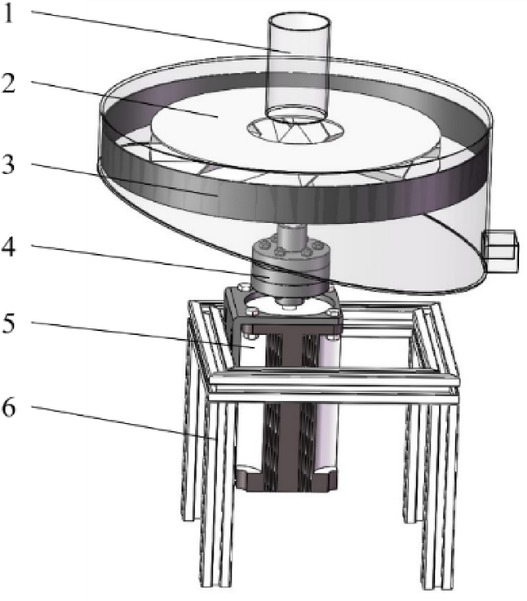
Structural diagram of centrifugal pellet impact device. (1) shell (2) impeller (3) annular collision plate (4) coupling 5.motor 6.rack.

To investigate dynamic breakage behavior, a centrifugal impact device was developed based on previously reported pellet attrition testers^30,31^. The apparatus consists of an impeller, annular impact plate, housing, and motor (Fig. 5). Pellets are accelerated radially by the rotating impeller and subsequently collide with the impact plate, where breakage occurs. Fragments are collected for sieving and mass-based analysis. Key structural parameters include: impeller diameter 300 mm, impact plate diameter 400 mm, housing inner diameter 402 mm, and plate clearance 30 mm. 150 g pellets were used for each test.

**Fig. 6:**
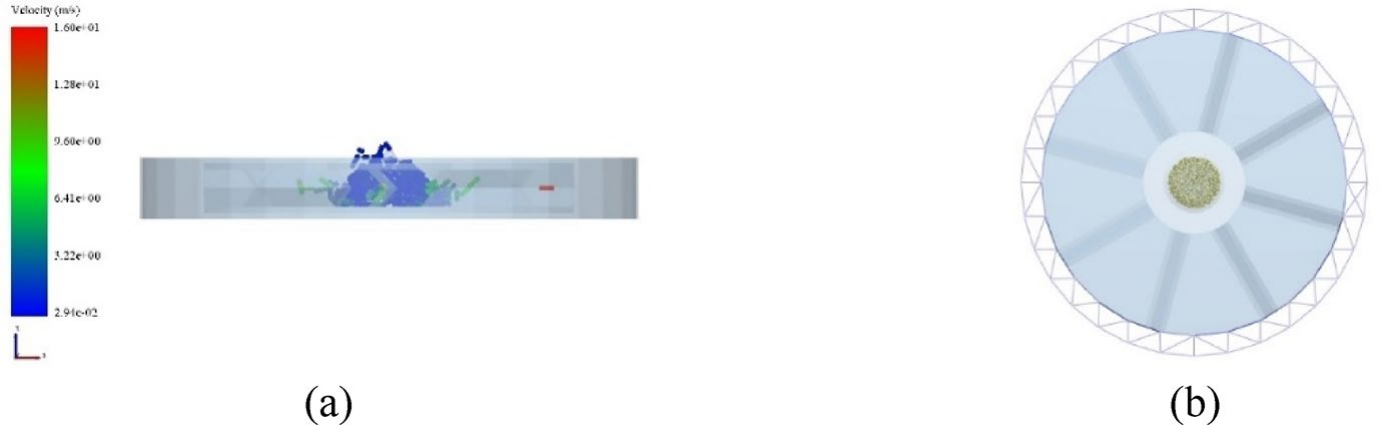
Simulation model of impeller in parameter optimization test. **(a)** Simplified model; **(b)** Grid area.

Breakage behavior was evaluated at impeller speeds of 500–1500 rpm and impact angles of 60°, 75°, and 90°. The DEM geometry was simplified while retaining essential kinematic features (Fig. 6). To monitor the pellet velocity at the impeller outlet, a circular Grid Bin region was defined, as illustrated in Fig. 6(b). The time step was set to 4.00 × 10^− 7^ s, with a total simulation duration of 0.40 s and a data save interval of 0.01 s. The pellet assembly consisted of 300 pellets, corresponding to 90,000 discrete particles and 246,000 contacts. A circular particle factory with a diameter of 40 mm was positioned above the impeller to represent the feeding process.

Other parameters used in simulation are listed in Table 3.

**Table 3:** Simulation parameters in EDEM.

Parameter	Value
Coefficient of restitution between particles	0.57
Coefficient of static friction between particles	0.33
Coefficient of rolling friction between particles	0.17
Coefficient of restitution between particle and wall	0.79
Coefficient of static friction between particle and wall	0.26
Coefficient of rolling friction between particle and wall	0.13

## Results and discussion

### Calibration of bonding parameters

#### Response surface test

Based on the determined parameter ranges, uniaxial compression simulations of the pellet feed were conducted. The experimental design matrix and corresponding simulation results are summarized in Table 4.

**Table 4:** Response surface test design and results of bonding parameters.

NO.	x_1_/(*N*/m^3^)	x_2_/(*N*/m^3^)	Breakage force/*N*
1	−1	−1	17.37
2	1	−1	42.10
3	−1	1	35.10
4	1	1	75.00
5	−1.414	0	9.65
6	1.414	0	65.20
7	0	−1.414	27.00
8	0	1.414	64.20
9	0	0	47.70
10	0	0	46.10
11	0	0	46.80
12	0	0	46.90
13	0	0	45.80

A comparison of different mathematical fitting models indicated that a full quadratic model provided the best fit. The analysis of variance (ANOVA) results is presented in Table 5. The model was highly significant (*P* < 0.0001), with a coefficient of determination R^2^ = 0.9937 and an adjusted R^2^ = 0.9891. The factors *x*_1_, *x*_2_, *x*_1_* × *_2_, and *x*2 1 all exhibited significant effects on the breakage force. These results demonstrate that the regression model provides an excellent fit and can be reliably used for subsequent analysis.

**Table 5:** Quadratic full model analysis of variance.

Source	Sum of squares	df	Mean square	F	*P*
Model	4087.74	5	817.55	200.77	< 0.0001
*x*_1_	2562.91	1	2562.91	629.39	< 0.0001
*x*_2_	1332.28	1	1332.28	327.18	< 0.0001
*x*_1_ *x*_2_	57.53	1	57.53	14.13	0.0071
*x*_1_^2^	134.53	1	134.53	33.04	0.0007
*x*_2_^2^	0.6685	1	0.6685	0.1642	0.6974
Residual	28.50	7	4.07		
Pure error	2.21	4	0.5530		
Cor total	4116.24	12			

**Fig. 7:**
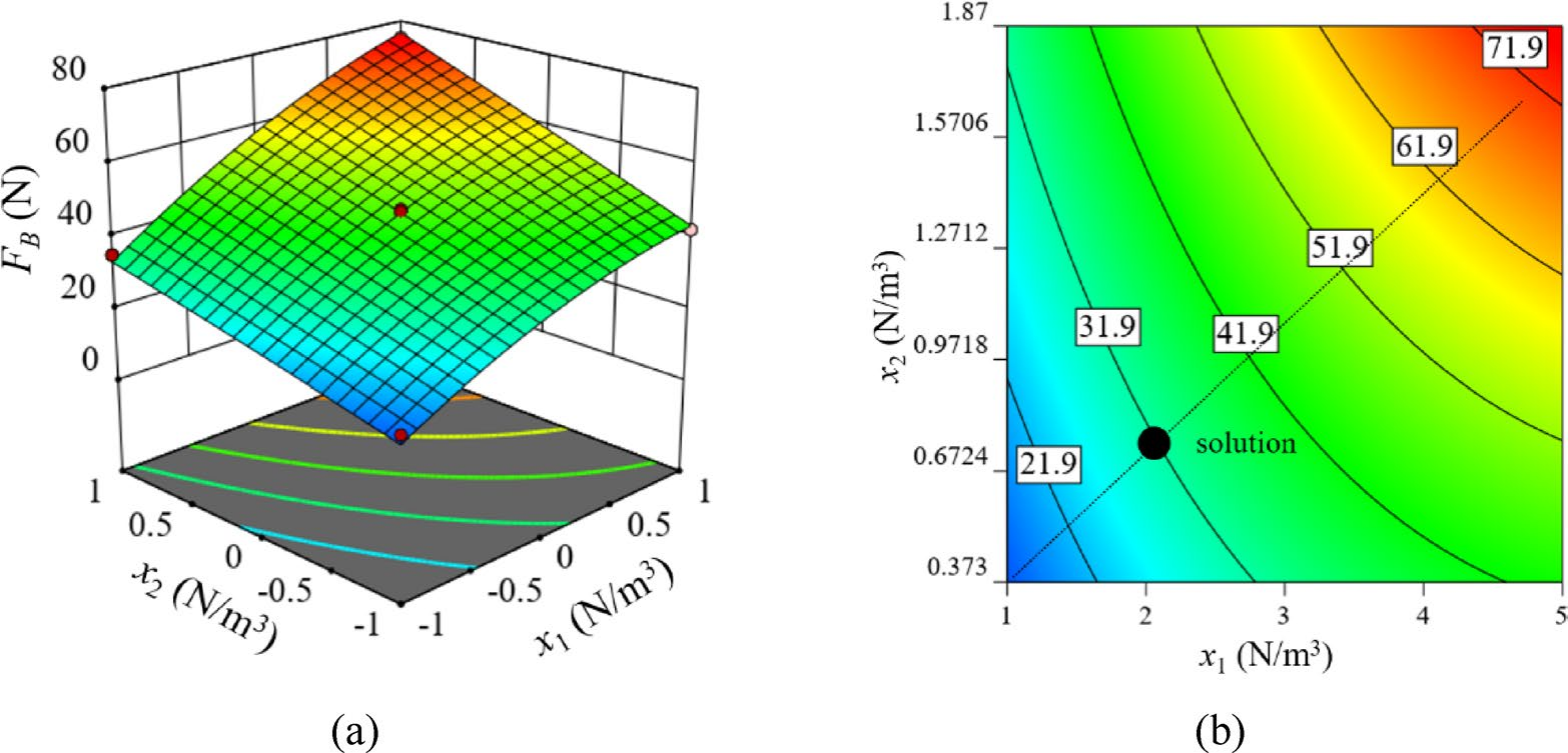
Effects of bonding parameters on breakage force. **(a)** 3D surface; **(b)** 2D surface.

The response surface describing the relationship between bonding stiffness parameters and breakage force (*F*_*B*_) is presented in Fig. 7. Both the three-dimensional and contour plots indicate a monotonic increase in breakage force with increasing normal stiffness (*x*_1_) and tangential stiffness (*x*_2_). When either parameter is held constant, the response increases consistently with the other, confirming the dominant and coupled influence of bond stiffness on macroscopic strength.

According to the above analysis, the fitting equation is obtained as follows:

5 $${F_B}=47.70+17.90{x_1}+12.90{x_2}+3.79{x_1}{x_2} - 4.92x_{1}^{2}$$

The quadratic regression equation was solved with the target value set to the experimentally measured breakage force of 32.3 N. Combined with the relationship between normal stiffness and tangential stiffness, the optimal values for *x*_1_ and *x*_2_ were determined to be 2.00 × 10^10^ N/m^3^ and 7.41 × 10^9^ N/m^3^, respectively.

**Fig. 8:**
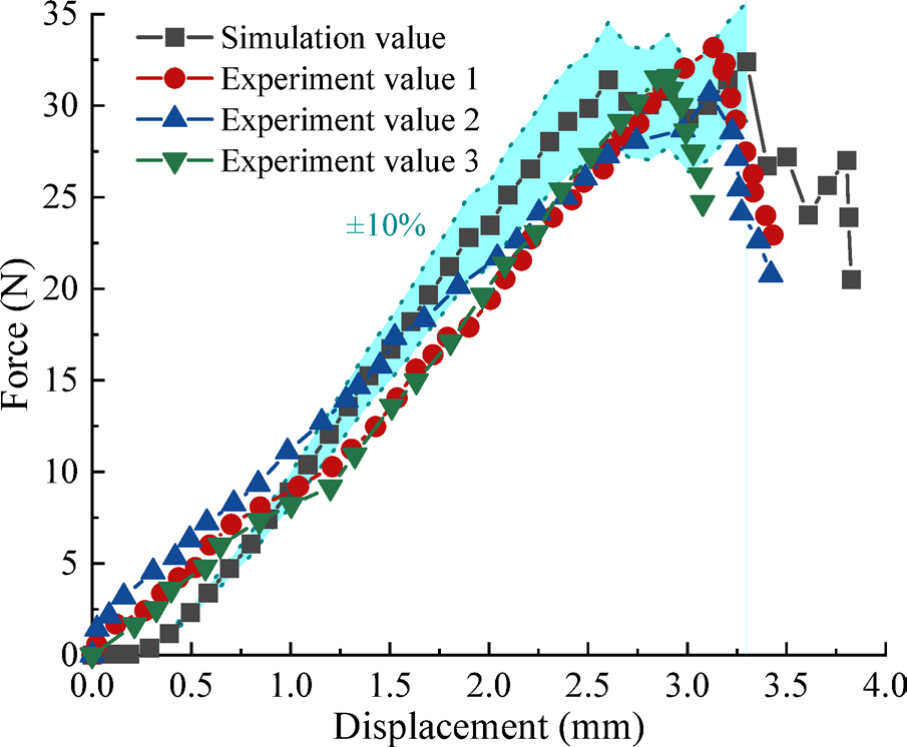
Comparison of the experiments and simulation results.

To further validate the calibrated parameter set, simulated force–displacement curves were compared with experimental results (Fig. 8). A ± 10% error band was introduced to quantify the deviation. In the initial loading stage (0–1.00 mm displacement), discrepancies exceed 10%, whereas beyond 1.0 mm the simulated curve remains within ± 10% of the experimental data, indicating improved agreement after the initial compaction phase. Fracture initiation in the simulation occurred at a displacement of 3.30 mm with an effective stiffness of 12.35 N/mm, while experimental fracture occurred at 3.05 mm with a stiffness of 10.29 N/mm. The relative error in fracture displacement was 7.58%, demonstrating satisfactory agreement in failure onset. This difference is mainly attributed to the inelastic deformation of real pellets during early compression, which is associated with the closure of microcracks, pore compaction, and internal structural rearrangement^32,33^.

#### Resolution and discretization sensitivity analysis

To assess the discretization dependence of the BPM, a systematic resolution sensitivity analysis was performed. The external geometry of the pellet was strictly preserved, while the internal discretization was varied by adjusting the sphere radius. Three representative resolutions were examined, comprising approximately 166, 300, and 582 spheres per pellet. The corresponding results are summarized in Table 6. Under uniaxial compression, all three models exhibited an identical macroscopic failure pattern: the pellet fractured into two dominant fragments, with crack initiation occurring at the bottom contact region followed by axial propagation. The predicted breakage force showed clear convergence, with a maximum relative deviation of 3.61% among the three pellets. Likewise, the absolute variation in bond breakage rate was limited to 2.62%, indicating that the global damage metric is weakly sensitive to particle resolution within the investigated range.

**Table 6:** Breakage behaviors of pellet feed at different particles.

Parameter	Simulation			Experiment
	166	300	582	
Fragment distribution				
Particle radius (mm)	0.48	0.40	0.32	
Contact radius (mm)	0.53	0.44	0.35	
Total bonding numbers	407	820	1520	
Bond breakage rate (%)	22.85	24.02	26.64	
Breakage force (N)	31.9	32.4	31.2	32.3

**Fig. 9:**
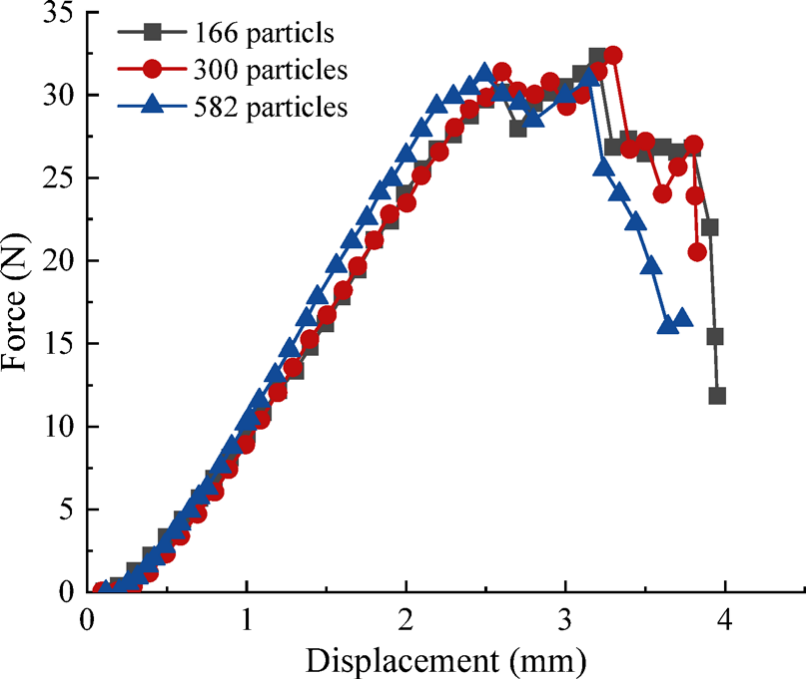
Comparison of the results at different pellets.

The force–displacement responses for the three resolutions are compared in Fig. 9. The curves exhibit substantial overlap in both the elastic regime and near peak load, further confirming numerical convergence. Considering the negligible differences in mechanical response and the substantially lower computational cost compared with the highest resolution model, the medium-resolution discretization was selected for subsequent simulations. This resolution provides a discretization-independent yet computationally efficient representation of pellet mechanical behavior.

### Simulation model application

In order to explore the breakage condition of pellet feed, the ratio of the number of broken bonds to the number of initial bonds was used to evaluate the breakage degree of pellet feed. The formula is as follows:

6 $$\delta {\mathrm{=}}\frac{{{N_{{\mathrm{breakage}}}}}}{{{N_{{\mathrm{initial}}}}}} \times 100{{\% }}$$

where *δ* is the breakage rate, %; *N*_breakage_ is the number of broken bonds; *N*_initial_ is the initial number of bonds generated. This value reflects the accumulation of internal damage within the pellet and is used as a microscopic indicator to evaluate relative breakage severity under different impact conditions.

#### Particle trajectory

**Fig. 10:**
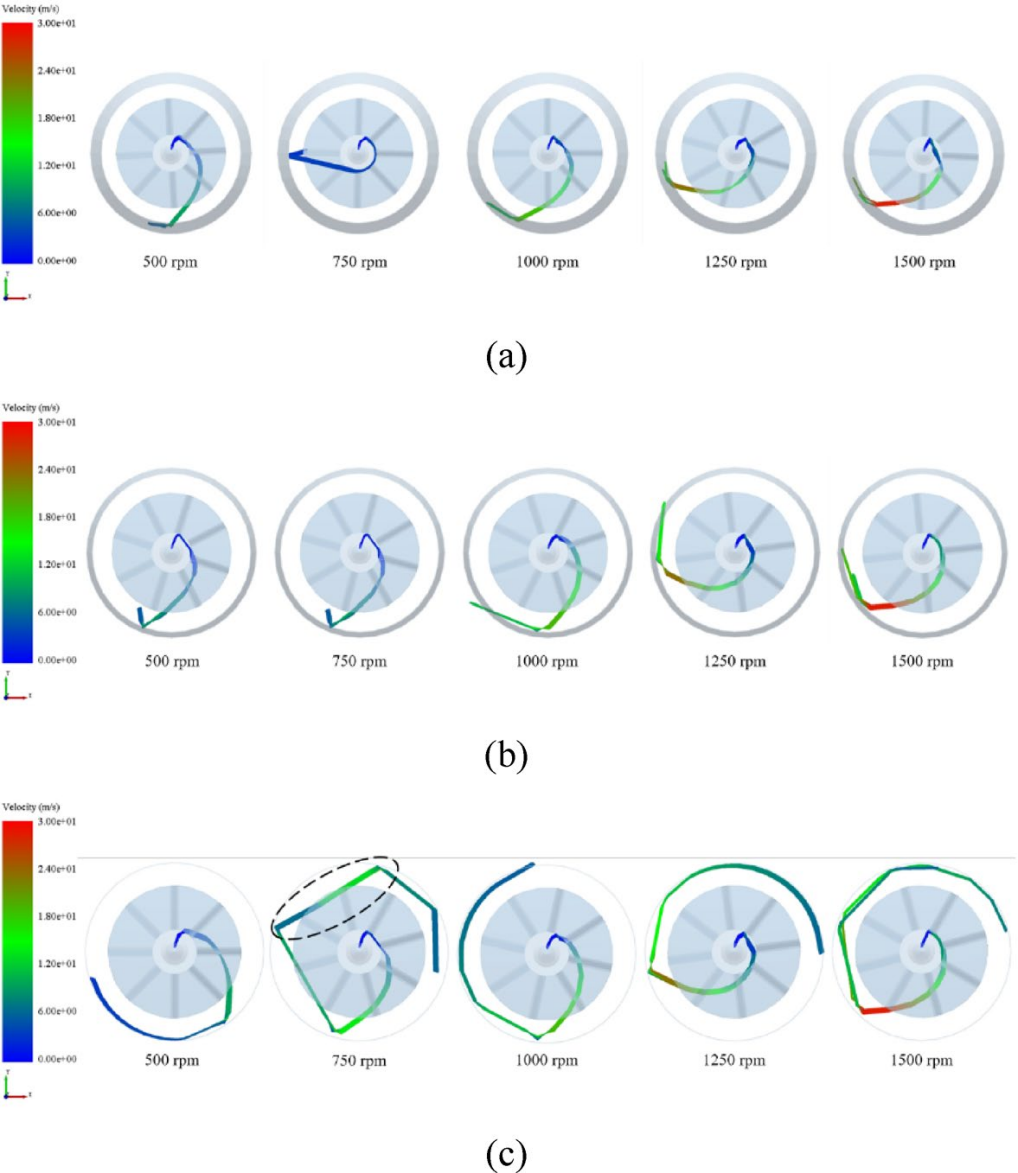
Particle trajectory of pellet feed under different parameters. **(a)** Impact angle = 60°; **(b)** Impact angle = 75°; **(c)** Impact angle = 90°.

A representative pellet was randomly selected to analyze its motion trajectory under different impeller rotational speeds and impact plate angles, as shown in Fig. 10. During impeller rotation, the pellet is simultaneously subjected to centrifugal and Coriolis forces, resulting in a smooth, curved trajectory within the impeller. After colliding with the wall, the pellet continues to move along the wall surface along an arc-shaped path. As the impact plate angle decreases, the internal trajectory length shortens, indicating that the pellet descends more rapidly after impact and exits the computational domain at an earlier stage. Notably, when the impact angle is 90°, a secondary impact event is observed, as indicated by the black dashed line.

#### The effect of impeller speed on particle breakage

**Fig. 11:**
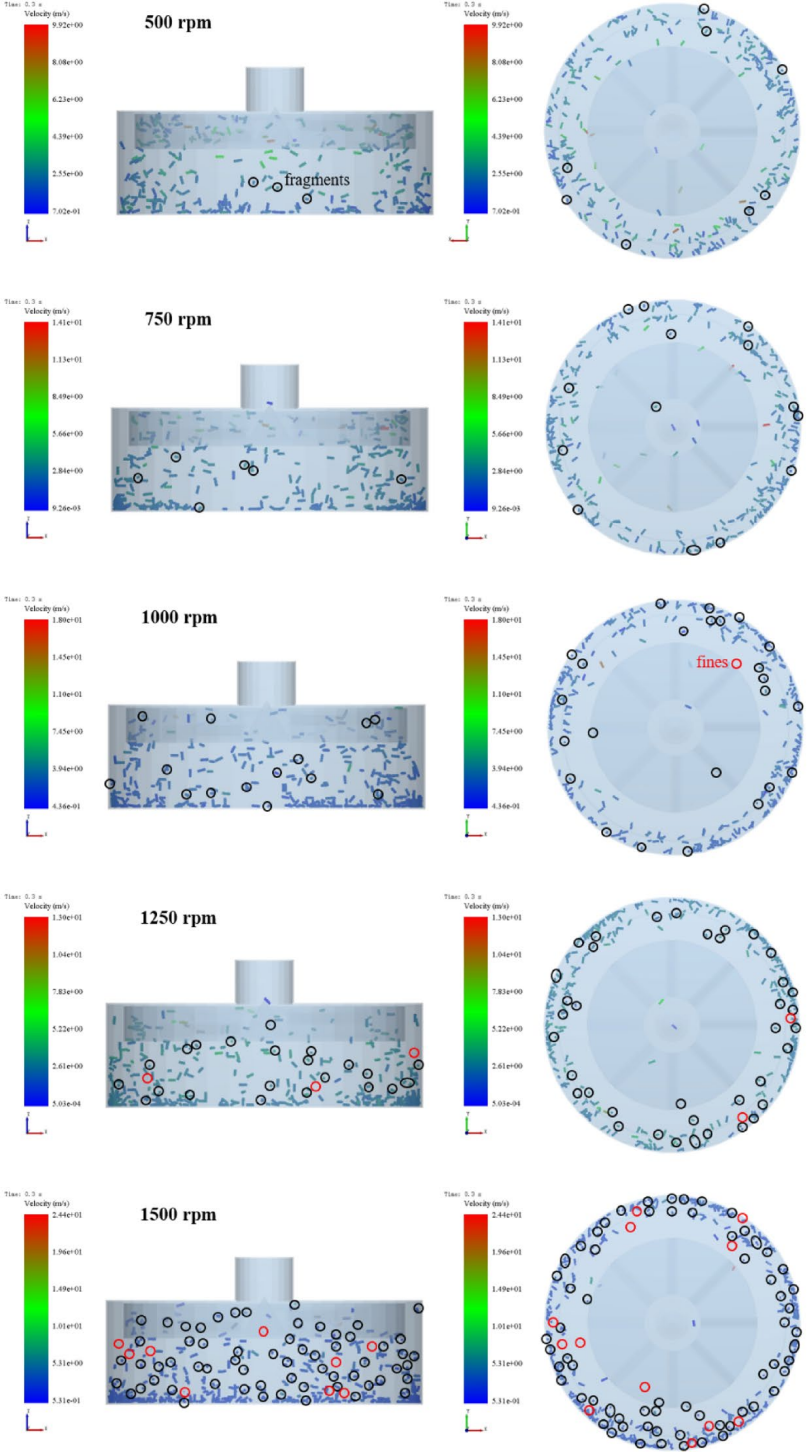
The breakage state of pellet feed under different impeller speeds.

To quantify pellet fragmentation, a hollow cylindrical monitoring region (diameter 450 mm, height 150 mm) was defined outside the impeller to statistically capture bond breakage events. The fragmentation states at identical simulation times under different impeller speeds are presented in Fig. 11. As the impeller speed increases, pellet damage becomes progressively more severe, characterized by a higher number of fragments and fines. The corresponding bond breakage rates at 500, 750, 1000, 1250, and 1500 rpm were 3.22%, 6.12%, 10.02%, 14.23%, and 21.43%, respectively, revealing a clear monotonic increase with rotational speed. This behavior arises from the higher impact velocities generated at elevated speeds, which increase the kinetic energy transferred to pellets upon collision, thereby intensifying crack propagation and fragmentation.

To validate the numerical results, centrifugal impact experiments were conducted under identical operating conditions. The experimentally measured breakage rates are summarized in Table 7. Overall, good agreement is observed between simulation and experiment, particularly at medium and high speeds. The relatively larger deviation at 500 rpm (20.50%) occurs within a low-damage regime. Under such low-energy conditions, the DEM model records internal bond failures that represent microcrack formation, even when these micro-damage events do not produce detachable fragments detectable by sieve-based mass measurements. Since the experimental breakage rate reflects only the mass of particles passing through the sieve, minor internal cracking without material detachment may not be captured. This difference in damage definition explains the slightly higher simulated breakage rate at low rotational speeds. The experimental standard deviations (0.21–0.55%) confirm good repeatability and reliability of the centrifugal impact tests.

**Table 7:** Breakage rate of pellet feed under different impeller speeds.

Impeller speed (rpm)	Breakage rate-simulation (%)	Breakage rate-experiment (%)	Relative error (%)
500	3.17	2.52 ± 0.21	20.50
750	6.05	5.13 ± 0.37	15.21
1000	10.02	9.09 ± 0.23	9.28
1250	14.23	14.06 ± 0.54	1.19
1500	21.43	20.17 ± 0.55	5.88

**Fig. 12:**
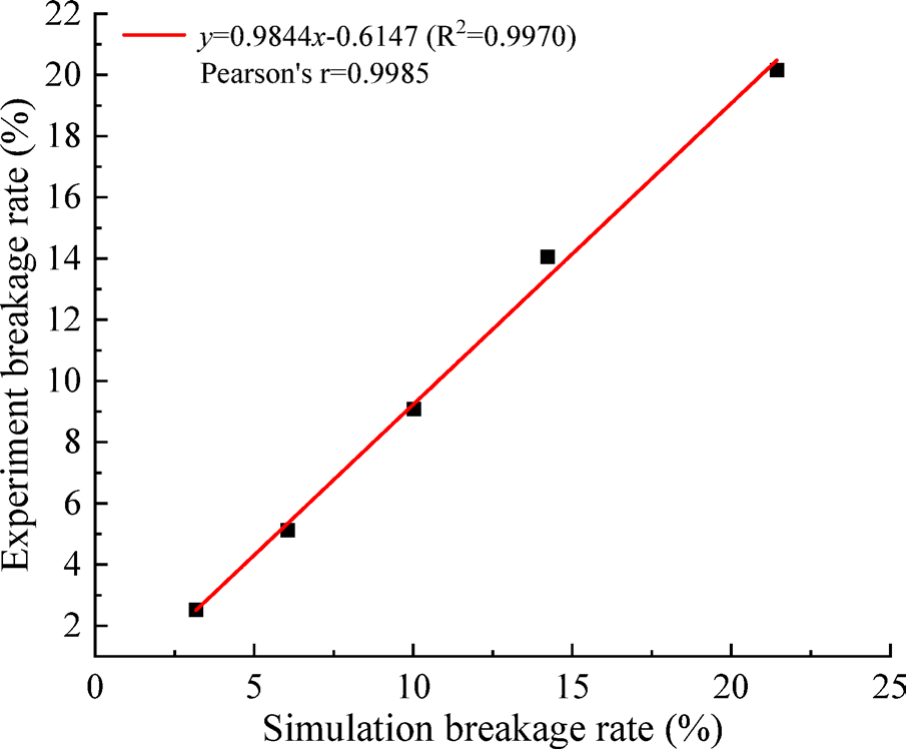
Correlation analysis between experiment and simulation.

To further clarify the physical meaning of bond breakage percentage, a correlation analysis was performed between the simulated bond breakage rate and the experimentally measured mass-based breakage rate under different impeller speeds. As shown in Fig. 12, a strong positive linear relationship was observed between the two variables. The coefficient of determination (R^2^) reached 0.9970, indicating that bond breakage percentage can effectively represent the macroscopic breakage trend.

#### The effect of impact angle on particle breakage

**Fig. 13:**
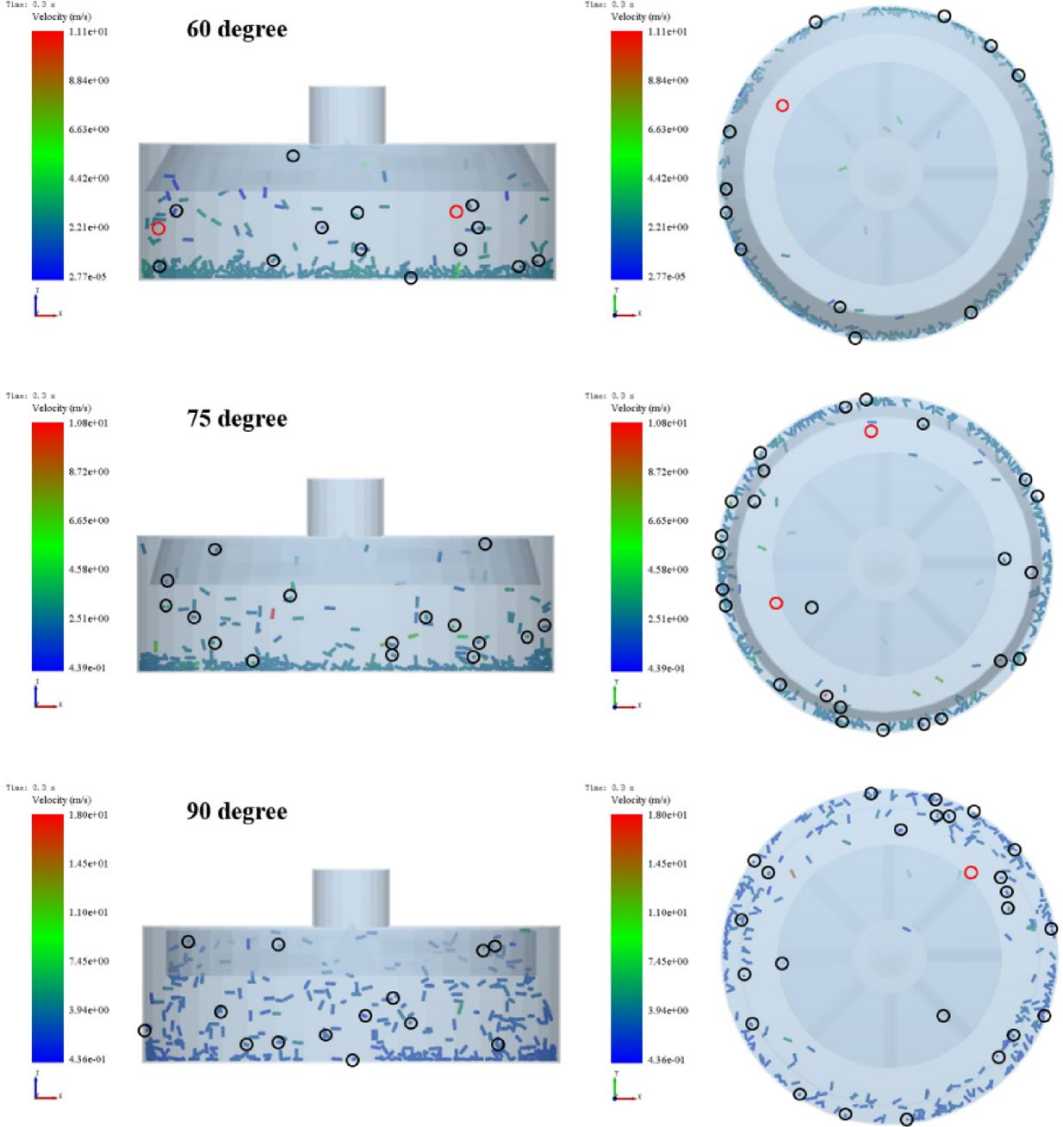
The breakage state of pellet feed under different impact angles.

To investigate the influence of impact angle on pellet breakage, the fragmentation state at a consistent time point was analyzed and the breakage rate was quantified under different impact angles. Taking an impeller speed of 1000 rpm as a representative case, the corresponding pellet fragmentation patterns are shown in Fig. 13. As the impact angle decreases, pellets tend to accumulate at the bottom of the housing after colliding with the impact ring, which significantly reduces the probability of re-entering the impeller for secondary acceleration. The calculated breakage rates were 5.99%, 6.86%, and 6.53% for impact angles of 60°, 75°, and 90°, respectively. These results indicate a non-monotonic dependence of breakage rate on impact angle, with a maximum observed at 75°.

#### Bond states

**Fig. 14:**
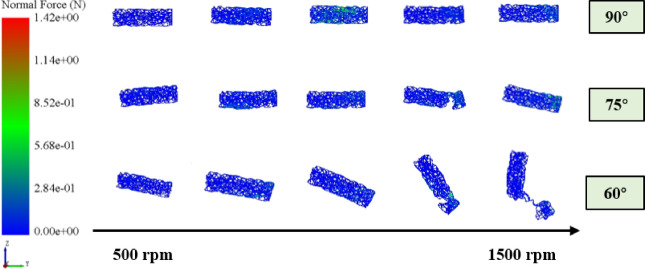
Bond states of pellet feed under impact.

To characterize the breakage behavior of pellet feed directly, the bond states of individual pellets were obtained, as shown in Fig. 14. It shows that as the impact angle decreases, the overall integrity of the pellet declines, while its rotational angle increases significantly. This phenomenon can be explained through the loading composition and energy dissipation mechanisms under different impact angles.

At an impact angle of 90°, the pellet is subjected primarily to axial loading, which leads to severe compression and rapid propagation of primary cracks. However, the loading duration is short, and the input energy may be dissipated through elastic collision; At an impact angle of 75°, the pellet experiences both significant axial and radial loadings, resulting in the peak value of breakage rate. The loading state not only creates a complex stress condition but also prolongs the effective contact and loading duration; As the impact angle decreases further, the radial loading becomes dominant. Most of the impact energy is converted into rotational kinetic energy and friction work. As a result, the breakage rate of pellet feed declines.

### Discussion

In the bonded particle model, bond breakage percentage quantifies internal micro-damage accumulation rather than direct mass-based fragmentation. While a strong linear correlation with experimentally measured breakage rate was established, the two metrics differ in physical meaning: bond failure reflects microscale cohesion loss, whereas experimental measurements capture only detached fragments passing through the sieve. Therefore, bond breakage percentage should be interpreted as a mechanistic indicator of internal damage governing macroscopic failure.

Model calibration was conducted under strictly controlled moisture conditions to ensure consistency between simulations and experiments. Although the calibrated parameters reliably reproduce breakage behavior within this range, moisture content significantly influences liquid bridge forces, inter-particle adhesion, and bulk stiffness. Substantial deviations in moisture may alter contact mechanics and fragmentation response.

Accordingly, extrapolation of the present parameter set beyond the tested moisture range should be undertaken with caution. Incorporating moisture-dependent contact formulations or performing systematic calibration across broader moisture levels would further enhance the general applicability of the proposed framework.

## Conclusion

A bonded particle model (BPM) for piglet pellet feed was developed, calibrated, and validated against uniaxial compression experiments, and subsequently applied to analyze impact-induced breakage in a centrifugal attrition device. The main conclusions are as follows:

(1) The bonding parameters were successfully calibrated, yielding a normal stiffness per unit area of 2.00 × 10^10^ N/m^3^, a tangential stiffness per unit area of 7.41 × 10^9^ N/m^3^, and both the normal and shear strengths of 500 MPa. The model accurately reproduced the experimental breakage force and force-displacement response.

(2) The breakage rate increased monotonically with impeller speed from 500 to 1500 rpm, reaching 21.43% at the highest speed. Simulation results were in good agreement with experimental measurements. A strong linear correlation (R^2^ = 0.9970) between bond breakage fraction and mass-based breakage rate demonstrates that bond failure percentage is a reliable microscopic indicator of macroscopic fragmentation.

(3) The impact angle exerted a non-linear influence on pellet breakage. As the angle increased from 60° to 90°, the breakage rate first increased and then decreased, with a maximum value of 6.86% at 75°. This trend reflects the combined effect of axial and radial loading components and the associated energy dissipation mechanisms.

## Data Availability

The datasets used and/or analysed during the current study available from the corresponding author on reasonable request.
